# Supplementary material for: Advanced glycation end-product crosslinking activates a type VI secretion system phospholipase effector protein
Source: Nat Commun. 2024 Oct 11;15:8804. doi: 10.1038/s41467-024-53075-x (PMC11470151; doi:10.1038/s41467-024-53075-x)
Supplement: Supplementary file 1 — Supplementary Information [file 41467_2024_53075_MOESM1_ESM.pdf]

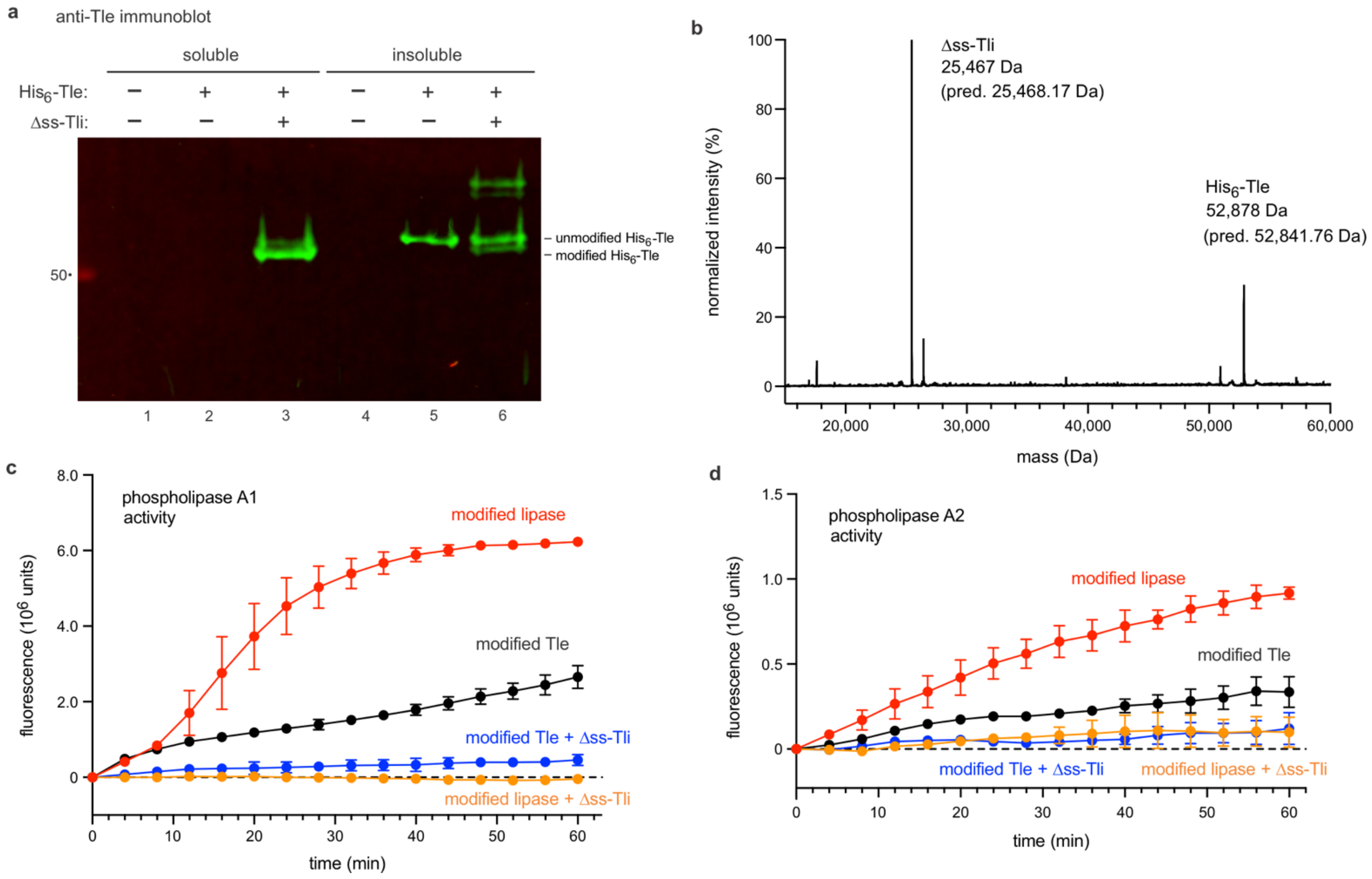

Supplementary Figure 1. Full-length Tle is modified. (a) His$_6$-Tle was produced with (or without) Δss-Tli immunity protein in *E. coli* and the cells broken by French press for fractionation into soluble and insoluble fractions by centrifugation. Fractions were analyzed by immunoblotting with polyclonal antibodies to Tle. Molecular mass markers (kDa) are on the left. This experiment was performed independently three times with similar results. (b) Deconvoluted mass spectrum of the purified His$_6$-Tle•Δss-Tli complex. (c) Modified His$_6$-Tle and His$_6$-lipase were isolated from Δss-Tli using Ni^{2+}-affinity chromatography under denaturing conditions, then refolded by dialysis for phospholipase A1 activity assays. (d) Phospholipase A2 activity assay of purified His$_6$-Tle and His$_6$-lipase. Phospholipase activity data are presented as mean values ± standard deviation for three independent experiments. Source data are provided as a Source Data file.

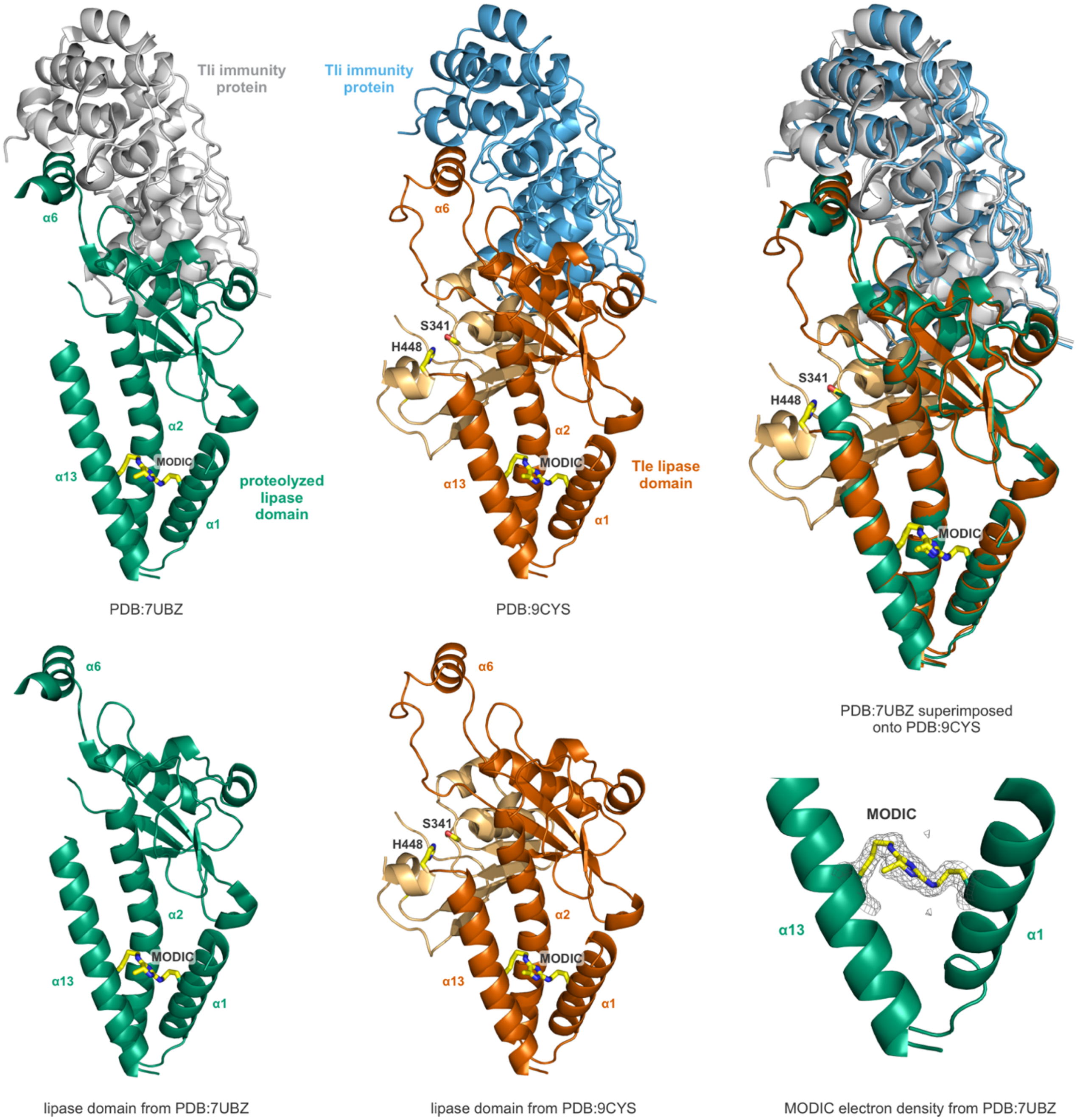

Supplementary Figure 2. Crystal structure of chymotrypsin-treated lipase•Δss-Tli complex. The structure of the proteolyzed complex (PDB:7UBZ) [https://www.rcsb.org/structure/7UBZ] is presented together with the native complex (PDB:9CYS) [https://www.rcsb.org/structure/9CYS]. The digested lipase segment is depicted in gold in structure 9CYS. Images of the isolated lipase domains and a structure superimposition are shown for comparison. MODIC electron density for the proteolyzed complex is illustrated by omit map at 3 sigma.

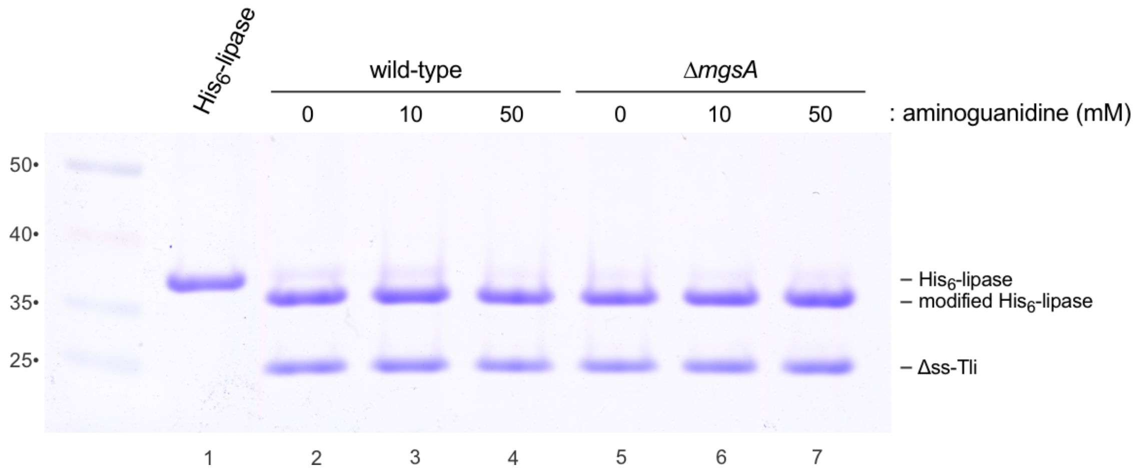

Supplementary Figure 3. Aminoguanidine treatment and deletion of methylglyoxal synthase (*mgsA*) does not affect lipase domain crosslinking *in vivo.* His_6-lipase was produced with Δss-Tli in wild-type and Δ*mgsA* cells treated with aminoguanidine. The His_6-lipase•Δss-Tli complex was purified by Ni^{2+}-affinity chromatography under non-denaturing conditions and analyzed by SDS-PAGE. Molecular mass markers (kDa) are on the left.

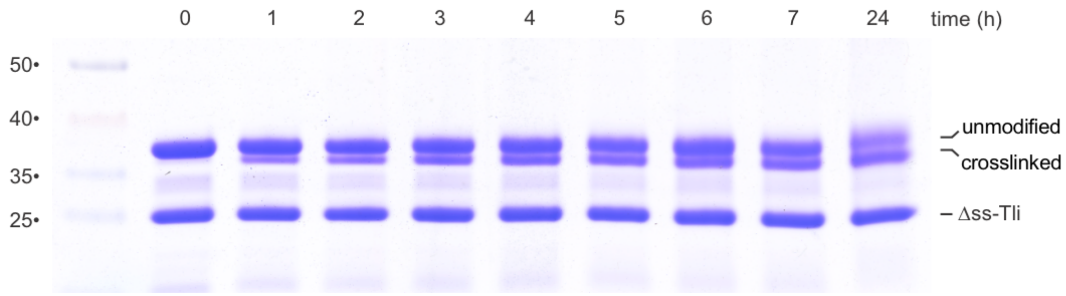

Supplementary Figure 4. *In vitro* methylglyoxal crosslinking time course. Unmodified His_6-lipase was purified and mixed with Δss-Tli *in vitro* to form a complex. The complex was incubated with 1 mM methylglyoxal at 37 °C for the indicated times and analyzed by SDS-PAGE. Molecular mass markers (kDa) are on the left.

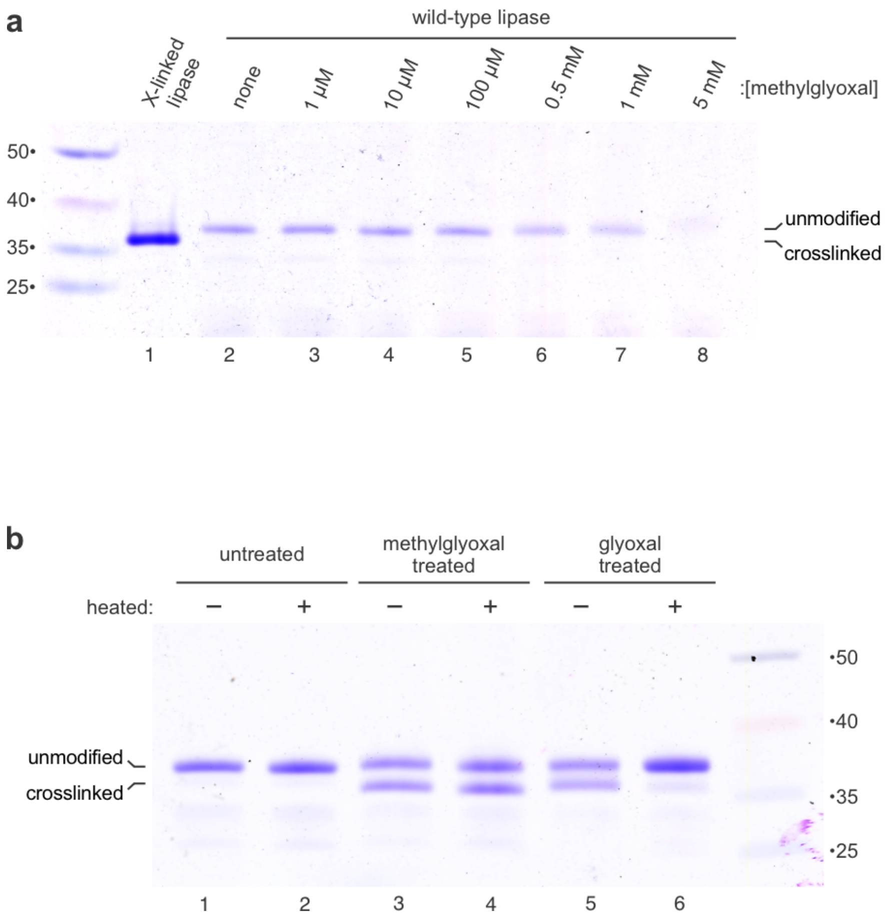

Supplementary Figure 5. *In vitro* crosslinking of the Tle lipase domain. (**a**) Unmodified His_6-lipase was incubated with methylglyoxal at the indicated concentrations for 7 h at 37 °C, then analyzed by SDS-PAGE. Molecular mass markers (kDa) are on the left. (**b**) The stability of methylglyoxal and glyoxal crosslinked His_6-lipase was assessed by SDS-PAGE analysis of unheated and 95 °C heated samples. Molecular mass markers (kDa) are on the right.

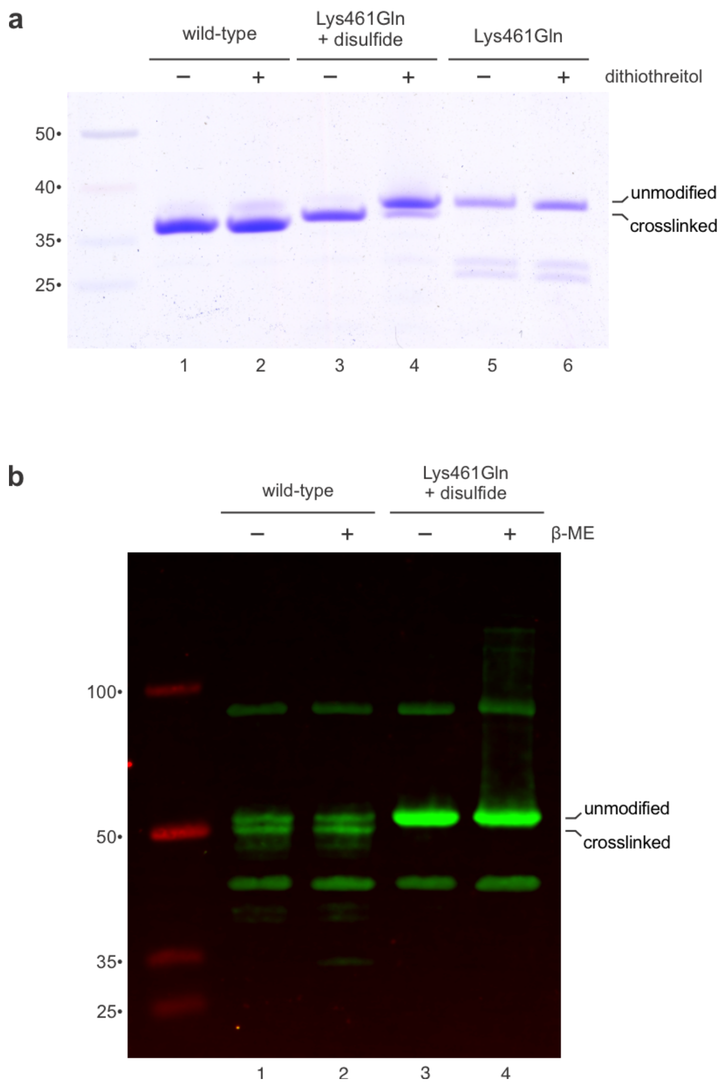

Supplementary Figure 6. Analyses of disulfide containing lipase domain. (**a**) The indicated His$_6$-lipase variants were produced with Δss-Tli in *E. coli* cells, then isolated by Ni^{2+}-affinity chromatography under denaturing conditions. Purified His$_6$-lipases were reduced with dithiothreitol for SDS-PAGE analysis and phospholipase A1 activity assays shown in Fig. 6d. Molecular mass markers (kDa) are on the left. (**b**) Tle-Tli and Tle(Lys461Gln-disulfide)-Tli production was induced with arabinose in *E. cloacae* cells. Urea-soluble total protein was extracted for immunoblot analysis using anti-Tle polyclonal antisera. Where indicated, samples were reduced with β-mercaptoethanol (β-ME). Molecular mass markers (kDa) are shown on the left. This experiment was performed independently three times with similar results.

Supplementary Table 1. Structural homologs of the Tle lipase domain.

Homolog	***PDB identifier***	***Z-score***	***rmsd (Å)***[a]	***lali***[b]	***nres***[c]	***% identity***[d]
Capsicum annum phospholipase A1	7X0D	13.3	3.8	194	386	15
Arabidopsis thaliana phospholipase A1	2YIJ	13.2	3.9	193	390	15
Aspergillus niger feruloyl esterase A	2IX9	12.7	3.4	186	386	15
Rhizopus chinensis triacylglycerol lipase	6A0W	12.7	3.7	173	287	16
Thermomyces lanuginosus lipase	4S0X	12.6	3.4	170	269	15
Aspergillus oryzae diacylglycerol lipase	5XK2	12.5	3.3	169	271	15
Penicillium roqueforti lipase	6L7N	12.3	3.2	170	270	12
Rhizomucor miehei triacylglycerol lipase	4TGL	12.3	3.3	172	265	20
Rhizopus niveus triacylglycerol lipase	1LGY	12.2	3.8	174	265	19
Penicillium cyclopium diacylglycerol lipase	5CH8	12.2	3.0	164	270	13
Rasamsonia emersonii lipase	6UNV	12.1	3.1	169	266	14
Yarrowia lipolytica triacylglycerol lipase	3O0D	12.0	3.6	171	296	15

[a]root-mean-square deviation over aligned α-carbon atoms
[b]number of residues in the structural alignment
[c]number of total residues in the homologous protein
[d]percent sequence identity between the aligned proteins

Supplementary Table 2 Bacterial strains and plasmids.

Strain	***Description***	***Reference***
ECL	*Enterobacter cloacae* subsp. *cloacae* ATCC 13047	ATCC
X90	*E. coli* F′ *lacI^q^ lac′ pro′/ara* Δ*(lac-pro) nal1 argE*(Am) *rif^r^ thi-1*, Rif^R	
CH2016	*E. coli* X90 (DE3) Δ*rna* Δ*slyD::kan*, Rif^R Kan^R	[58]
CH5691	*E. coli* X90 (DE3) Δ*rna* Δ*slyD* Δ*mgsA::kan*, Rif^R Kan^R	this study
CH6247	*E. coli* X90 (DE3) Δ*rna* Δ*slyD*, Rif^R	
CH11396	*E. cloacae* Δ*tssM*	[27]
CH11895	*E. cloacae* Δ*tle* Δ*tli::spc*, Spc^R	[27]
CH14384	*E. cloacae* Δ*tle* Δ*tli::spc rif*, Spc^R Rif^R	[12]
Plasmid		
pCH450	pACYC184 derivative with *E. coli araC* and *araBAD* promoter for arabinose-inducible expression, Tet^R	[58]
pCH495	pCH450K::*(*Δ*ss)tli*, Tet^R	[12]
pCH1382	pCH450::*his$_6$-lipase(K186C,C456S,K461Q,A472C)*, Tet^R	this study
pCH2199	pSH21::*tle*, Amp^R	this study
pCH2826	pSCBAD::*tle*, Tp^R	this study
pCH3128	pCH450::*tle-tli*, Tet^R	[12]
pCH3291	pET21P::*(*Δ*ss)tli,* Amp^R	this study
pCH3763	pCH450::*vgrG2-lipase(S341A)-tli*, Tet^R	[12]
pCH3923	pSCBAD::*tle(K186C,C456S,K461Q,A472C)-tli*, Tp^R	this study
pCH3936	pCH450::*his$_6$-lipase(S341A)*, Tet^R	this study
pCH4362	pSCBAD::*tle-tli*, Tp^R	this study
pCH5119	pSCBAD::*hchA*, Tp^R	this study
pCH5120	pSCBAD::*ydjG*, Tp^R	this study
pCH5121	pSCBAD::*yqhDE*, Tp^R	this study
pCH5501	pSCBAD::*tle(R180K)-tli*, Tp^R	this study
pCH5502	pSCBAD::*tle(Q459K)-tli*, Tp^R	this study
pCH5503	pSCBAD::*tle(Q460E)-tli*, Tp^R	this study
pCH5892	pSH21::*tle-tli*, Amp^R	this study
pCH7088	pCH450::*his$_6$-lipase*, Tet^R	this study
pCH7599	pCH450::*his$_6$-lipase(R180K)*, Tet^R	this study
pCH7600	pSCBAD::*tle(E458Q)-tli*, Tp^R	this study
pCH7601	pSCBAD::*tle(K461Q)-tli*, Tp^R	this study
pCH7620	pCH450::*his$_6$-tle*, Tet^R	this study
pCH7694	pSCBAD::*gloA*, Tp^R	this study
pCH7695	pMCSG63::*TEV-(*Δ*ss)tli*, Amp^R	this study
pCH8259	pCH450::*his$_6$-arfA*, Tet^R	[55]
pCH8717	pCH450::*his$_6$-lipase(Q459K),* Tet^R	this study
pCH8718	pCH450::*his$_6$-lipase(Q460E)*, Tet^R	this study
pCH8871	pCH450::*his$_6$-lipase(E458Q)*, Tet^R	this study
pCH8872	pCH450::*his$_6$-lipase(K461Q)*,Tet^R	this study
pCH8904	pSH21::*lipase(K186C,C456S,K461Q,A472C)*, Amp^R	this study
pCH14212	pCH450::*tle*, Tet^R	[12]
pCH15269	pSH21::*lipase*, Amp^R	[12]
pSCBAD	pBBR1 derivative that carries *araC* and P_{BAD} promoter, Tp^R	[57]
Abbreviations: Amp^R, ampicillin resistant; Kan^R, kanamycin resistant; Rif^R, rifampicin resistant; Spc^R, spectinomycin resistant; Tet^R, tetracycline resistant; Tp^R, trimethoprim resistant		

Supplementary Table 3. Oligonucleotides.

Identifier	*Description*	*Sequence*
CH943	ara-for	5′ - GAT TAG CGG ATC CTA CCT GAC GCT TTT TAT CGC
CH3719	tli-M24-Kpn-for	5′ - AAA GGT ACC ATG GAT TTA AAA CCA G
CH3419	tli-Xho-rev	5′ - ATA CTC GAG CTA TTT AAC CGG AGT TGG TG
CH4469	tle-Eco-for	5′ - AGG GAA TTC CGA ATG TAC AAC ATA AAA TTT GTC
CH4703	tle-Spe-for	5′- TTT ACT AGT ATG TAC AAC ATA AAA TTT GTC TAT CTT TTC AG
CH4762	tle-S341A-for	5′ - CGA TAT TGC AGG CCA CGC TCT GGG TGG TGG G
CH4763	tle-S341A-rev	5′ - CCC ACC ACC CAG AGC GTG GCC TGC AAT ATC G
CH5087	tle-T172-Spe-for	5′ - TTT ACT AGT ACC AAA GCT GAA CGC TGG C
CH5539	tle-H448A-for	5′ - GTC CAT TGG ACC GCG CTG GCA TTG GTC AGG
CH5675	tle-K461Q-Xho-rev	5′ - TTC TCG AGT TAT GCA CGA CTC CTA ATA ATT GAA ATG TCT TCA TCC TGT TGC TGT TCT ATG
CH5688	tle-R180K-Spe-for	5′ - CAC ACT AGT ACC AAA GCT GAA CGC TGG CAG GCG AAG AAG GAT CTG ATT GC
CH5689	tle-E458Q-for	5′ - GGC AAT AGA TTG CAT ACA ACA GCA AAA GGA TGA
CH5690	tle-461Q-for	5′ - GAT TGC ATA GAA CAG CAA CAG GAT GAA GAC ATT TCA A
CH5721	tle-R180K-for	5′ - CGC AAT CAG ATC CTT CTT CGC CTG CCA GCG TT
CH5722	tle-Q459K-for	5′ - GCA ATA GAT TGC ATA GAA AAG CAA AAG GAT GAA GAC
CH5723	tle-Q460E-for	5′ - ATA GAT TGC ATA GAA CAG GAA AAG GAT GAA GAC ATT TC
CH5751	ECL-tli-TEV-D25-Kpn-for	5′ - TTT GGT ACC GAG AAC CTG TAC TTC CAA TCC GAT TTA AAA CCA GAT AAT TAC TTT AGC GGA
CH5761	gloA-Eco-for	5′ - TTT GAA TTC ATG CGT CTT CTT CAT ACC ATG
CH5762	gloA-Xho-rev	5′ - TTT CTC GAG TTA GTT GCC CAG ACC G
CH5879	tle-E393A-for	5′ - CGT GTT GAA GGT GCA TTG CTG ACA AAA ATC C
CH5907	tle-K186C-for	5′- AAG GAT CTG ATT GCG TGT GGG AGT AAT AGC CTC
CH5908	tle-K186C-rev	5′ - GAG GCT ATT ACT CCC ACA CGC AAT CAG ATC CTT
CH5909	tle-A472C-Xho-rev	5′ - AAG CTC GAG TTC AAC AAC GAC TCC TAA TAA TTG AAA TGT C
CH5959	tle-C456S-for	5′ - GGT CAG GCA ATA GAT TCC ATA GAA CAG CAA
CH5960	tle-C456S-rev	5′ - TTG CTG TTC TAT GGA ATC TAT TGC CTG ACC
CH6062	tle-A472C-tli-for	5′ - ATT TCA ATT ATT AGG AGT CGT TGT TGA TGA AAT CGT TCT TAT CAG GCT GG
CH6063	tle-A472C-tli-rev	5′ - CCA GCC TGA TAA GAA CGA TTT CAT CAA CAA CGA CTC CTA ATA ATT GAA AT

CH6310	hchA-Mfe-for	5´ - AAG CAA TTG ACT ATG ACT GTT CAA ACA AGT AAA AAT CC
CH6311	hchA-Sbf-rev	5´ - AAC CCT GCA GGG ATT AAC CCG CGT AAG CTG CC
CH6312	ydjG-Eco-for	5´ - TAA GAA TTC CAA ATG AAA AAG ATA CCT TTA GGC
CH6313	ydjG-Pst-rev	5´ - CGT CTG CAG TAT TTA ACG CTC CAG G
CH6314	yqhD-Eco-for	5´ - AGG GAA TTC GTA ATG AAC AAC TTT AAT CTG
CH6315	yqhE-Pst-rev	5´ - GAA TCT GCA GGT TAG CCG CCG AAC TG
ZR248	tle-Xho-rev	5´ - TTT CTC GAG CTG ATA AGA ACG ATT TCA TGC ACG
